# Survival Outcomes of Patients With Mediastinal Germ Cell Tumors: Experience of a Cancer Center in South America

**DOI:** 10.3389/fonc.2021.758496

**Published:** 2022-01-03

**Authors:** Camilo Vallejo-Yepes, Carlos Andrés Carvajal-Fierro, Ricardo Brugés-Maya, Julian Beltrán, Ricardo Buitrago, Rafael Beltrán-Jimenez, José Alexander Carreño-Dueñas

**Affiliations:** ^1^ Clinical Oncology, National Cancer Institute, Universidad El Bosque, Bogota, Colombia; ^2^ Thoracic Surgery, National Cancer Institute, Bogota, Colombia; ^3^ Interventional Radiology, National Cancer Institute, Bogota, Colombia; ^4^ Research Department, National Cancer Institute, Bogota, Colombia

**Keywords:** mediastinal neoplasms, non-seminomatous germ cell tumor, seminoma, South America, Colombia

## Abstract

**Purpose:**

Mediastinal germ cell tumors (GCT) are rare neoplasms associated with poor survival prognosis. Due to their low incidence, limited information is available about this disease in South America. The objective of this study is to report the clinical characteristics and outcomes of patients with mediastinal GCT in a cancer center in Colombia.

**Materials and Methods:**

We conducted a retrospective analysis of patients with mediastinal GCT treated at the National Cancer Institute at Bogota (Colombia) between 2008 and 2020. Survival curves were presented using the Kaplan–Meier method. Chi-square and Cox proportional hazard model tests were used for data analysis.

**Results:**

Sixty-one patients were included in the study. Of them, 60 were male and 51 (83.6%) of whom had non-seminomatous germ cell tumors (NSGCT). Twenty-nine patients (47.5%) presented with superior vena cava syndrome, and 18 (29.5%) patients had extrapulmonary metastatic involvement. The three-year overall survival (OS) of NSGCT patients was 26%. The 3-year OS of NSGCT patients who underwent surgical resection of residual mediastinal mass after chemotherapy was 59%. Non-surgical management after first-line chemotherapy was associated with a worse survival prognosis in NSGCT patients (*p* = 0.002). Ten patients with mediastinal seminomatous germ cell tumors (SCGT) achieved a 3-year OS of 100%.

**Conclusion:**

Mediastinal NSGCT had poor outcomes. Surgery of the residual mass after first-line chemotherapy seems to improve the outcome of NSGCT patients. Advanced disease at presentation may reflect inadequate access to reference cancer centers in Colombia and potentially explain poor survival outcomes in this cohort. On the other hand, mediastinal SCGT is a biologically different disease; most patients will achieve disease remission and long-term survival with first-line chemotherapy.

## Introduction

Germ cell tumors (GCT) are one of the leading causes of cancer in men between the ages of 15 and 35 (1), with most of these neoplasms arising from testicular sites. However, in up to 5.7% of the patients, the tumor is localized in an extra-gonadal origin. In these cases, the anterior mediastinum constitutes the most frequent location (2). Mediastinal GCT shares histopathologic characteristics with their testicular counterpart; nonetheless, they usually tend to have a more aggressive biological behavior and worse survival outcomes (3, 4). Histology plays a major role in prognosis and treatment. Seminomatous germ cell tumors (SGCT) may reach a 5-year overall survival of over 90% and non-seminomatous germ cell tumors (NSGCT) almost 45% (4, 5). However, histology is not the only prognostic factor related to a lower probability of survival as other variables have been described. These include alpha-fetoprotein (AFP) or beta-human chorionic gonadotropin elevation after chemotherapy or incomplete tumor resection in the postoperative period, tumor viability in the pathology specimen after surgery, and induction chemotherapy (4–6).

Due to the poor outcomes reported, mediastinal NSGCTs are classified as with poor prognoses in international clinical practice guidelines (7). Furthermore, due to their low incidence, no randomized clinical trials are available to support a standard of care. Therefore, treatment is based on the management of gonadal primary tumors with poor prognosis, where the standard of care is cisplatin-based chemotherapy regimens and resection of any residual mass (8).

Limited information is available on the clinical characteristics and survival outcomes of mediastinal GCT in Colombia, and the inadequate access to health services in our country may likely lead to worse survival outcomes in these patients, as has been observed in other lower- or median-income countries (9). To improve the knowledge about this disease in our country, we performed a retrospective analytic study in a cancer center in Bogota, Colombia, by reporting the clinical characteristics and survival outcomes of patients with mediastinal GCT referred to our hospital over 12 years.

## Methods

### Study Population

**Figure 1:**
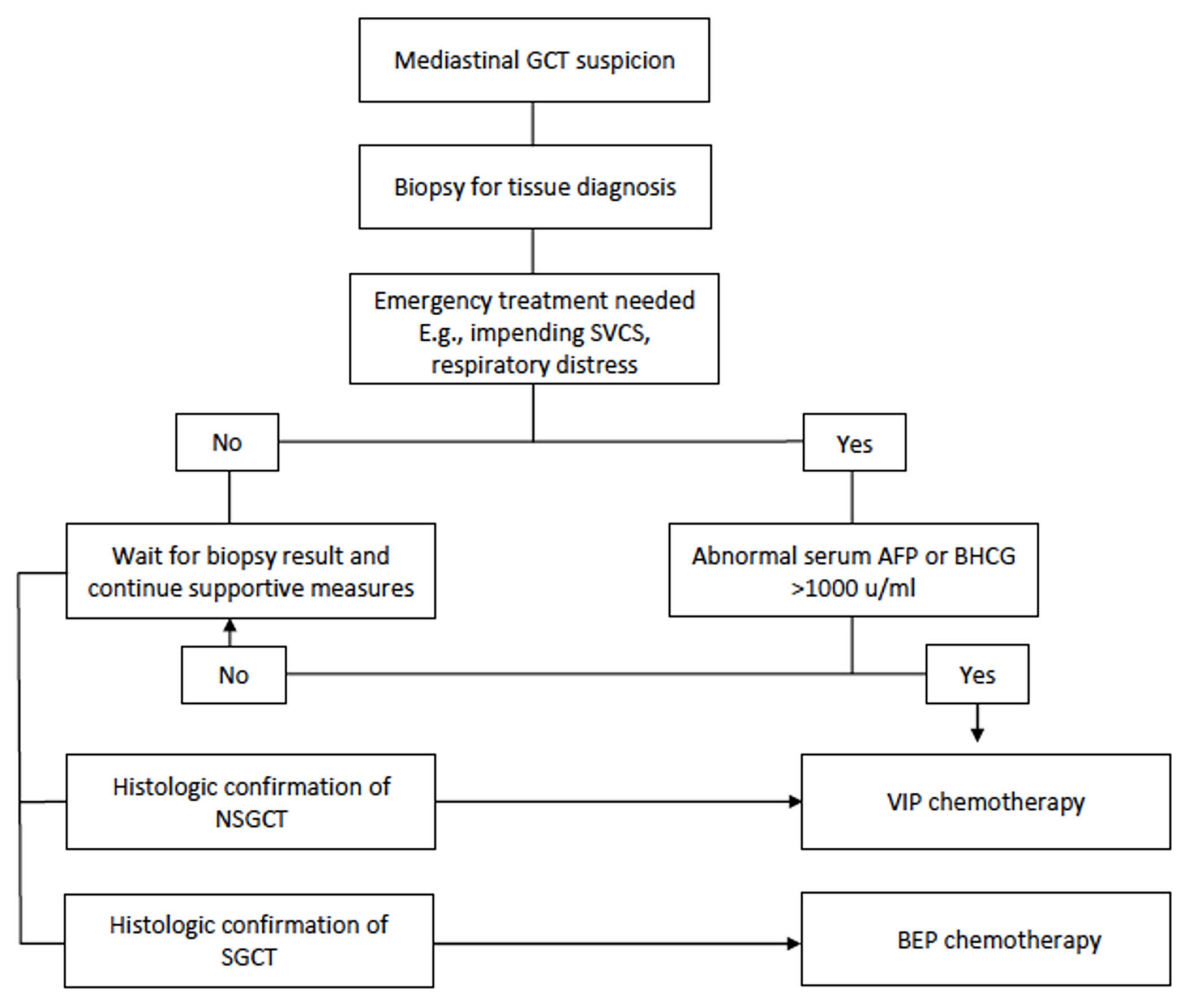
Initial approach of the patient with mediastinal germ cell tumor.

**Figure 2:**
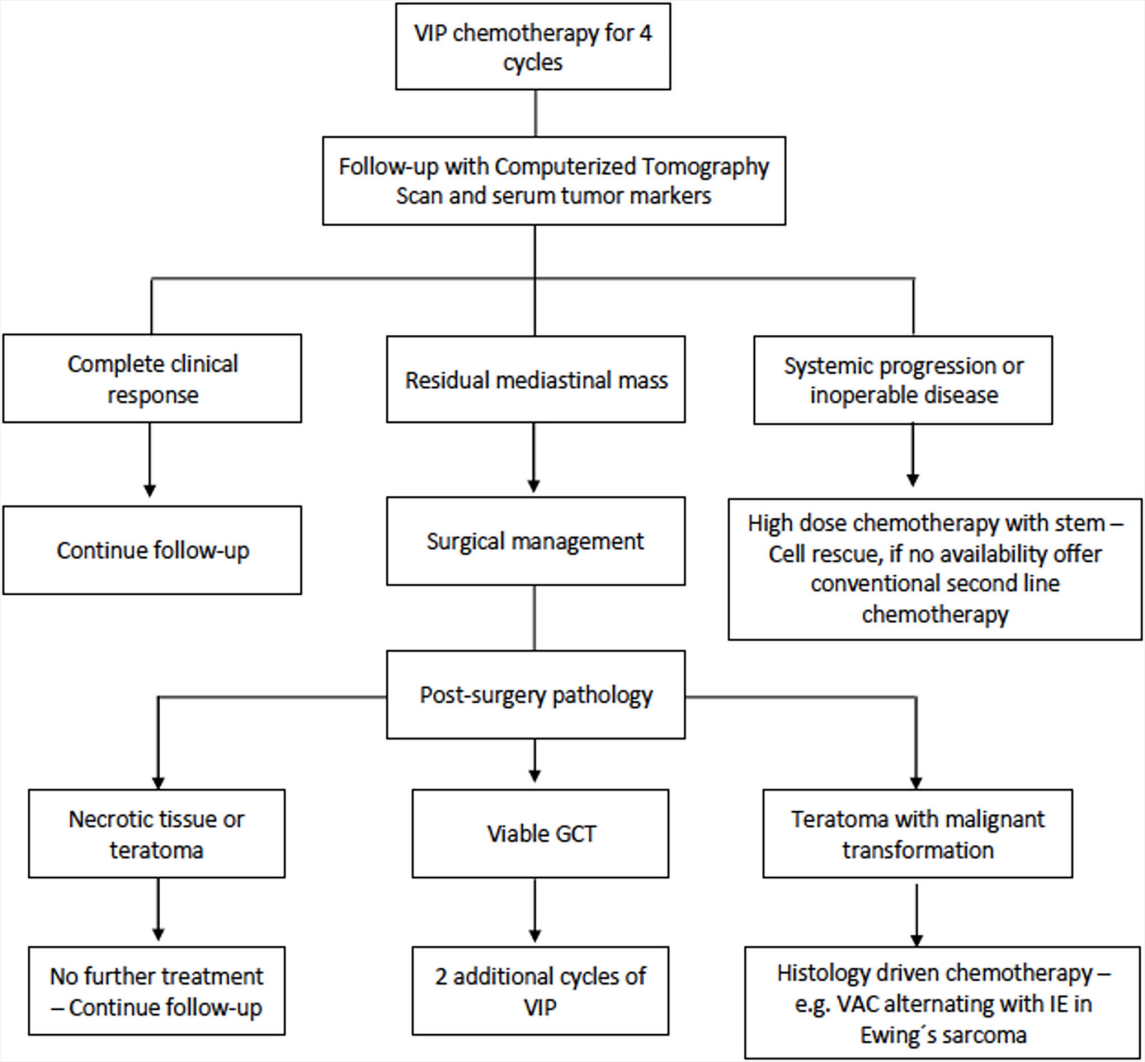
Subsequent management of mediastinal non-seminomatous germ cell tumor.

Patients with mediastinal GCT treated at the National Cancer Institute in Bogota (Colombia) between 2008 and 2020 were included in the study. Mediastinal GCT diagnosis was made using results from percutaneous biopsy, surgical specimen histopathologic analysis, or elevation of serum tumor markers which included AFP or beta-human chorionic gonadotropin in patients with an anterior mediastinal mass. Gonadal primary GCT was ruled out with clinical examination and a negative testicular/pelvic ultrasound. The initial approach to the patient with mediastinal GCT in our cancer center is shown in **Figure 1**. The subsequent management of mediastinal NSGCT is described in **Figure 2**. We determined the relative dose intensity (RDI) for the chemotherapy regimen by calculating the average of the RDI of every single agent in the regimen.

Medical records were reviewed, and the RedCap platform was used to collect data for demographic, clinical, laboratory, radiologic, histopathologic, surgical, and survival variables. The study was approved by the institutional ethics and research committee (no. CEI-00526-20) before collecting data of patients from medical records and was supervised by an independent clinical monitoring group.

### Statistical Analysis

The analysis was performed through the dynamic table function of SPSS (IBM SPSS Statistics for Windows, version 19.0, released 2010, IBM Corp., Armonk, NY, USA). Descriptive statistics were used to report the medians and range as well as minimum and maximum of continuous variables. Number and percentage were used to describe categorical variables. Survival curves were presented using the Kaplan–Meier method and compared using the log-rank test. Overall survival (OS) was defined as the time between diagnosis and the date of death. The confirmation of the vital status of each patient was made quarterly, and they were considered as censored data when the follow-up time of a patient ended before death or before completing the observation period or when the survival times could not be accurately established. Progression-free survival was defined as the time between treatment and radiologic confirmation of progression. The correlation between survival outcomes and other variables was accomplished using chi-square and Fisher’s exact test. *P*-values ≤0.05 were considered statistically significant. A multivariate analysis was performed with a Cox proportional hazard model and presented as hazard ratio (HR) and 95% confidence interval (CI).

## Results

### Clinical Characteristics

Between 2008 and 2020, 61 patients with mediastinal GCT were treated. The mean age was 24 years old (range, 13–52), and 60 patients were male. No patient had synchronous or metachronous hematologic neoplasms. Fifty-one patients had NSGCT, and 10 patients had SGCT. Regarding radiological characteristics, 29 patients (47.5%) presented with superior vena cava syndrome (SVCS). Five patients (17.2%) with NSGCT and SVCS underwent stent implantation. Visceral metastasis was observed in 23 (37.7%) patients; 18 (29,5%) of them had extrapulmonary metastasis, and 12 (19.6%) had pulmonary metastases (**Table 1**).

**Table 1:** Clinical characteristics.

Characteristics	*n* (%)
Male	60 (98.3)
Female	1 (1.7)
Histology	
Seminoma	10 (16,3)
Non-seminomatous	51 (83.6)
Mature or immature teratoma	10 (19.6)
Mixed germ cell tumors (GCT)	9 (17.6)
Endodermal sinus	7 (13.7)
Non-specified GCT	7 (13.7)
Teratoma with malignant differentiation	5 (9.8)
Embryonal carcinoma	2 (3.9)
No histological confirmation^a^	11 (21.5)
Tumor biomarkers	
Alpha-fetoprotein	
Normal	20 (32.7)
Abnormal	35 (57.3)
No data	6 (9.8)
Beta-human chorionic gonadotropin	
Normal	17 (27.8)
Abnormal	35 (57.3)
No data	9 (14.7)
Imaging characteristics	
Superior vena cava syndrome	29 (47.5)
Metastatic involvement	23 (37.7)
Extrapulmonary metastatic involvement	18 (29.5)
Pericardial effusion	3 (4.9)
Pleural effusion	2 (3.2)

a Abnormal alpha-fetoprotein levels and urgent clinical situation.

### Treatment

Fifty-five patients (90.2%) received first-line chemotherapy with the most common scheme [45 patients (73.8%) being bleomycin, etoposide, and cisplatin (BEP)]. This was followed by etoposide, ifosfamide, and cisplatin (VIP) in six patients (9.8%) and by paclitaxel, ifosfamide, and cisplatin (TIP) in two patients. One patient received therapy with carboplatin, etoposide, and bleomycin, and another one received monotherapy with etoposide. Pegfilgrastim was used with induction chemotherapy in 44 patients.

In the first-line setting, the RDI was 90% for the 45 patients undergoing BEP, 76% for the six patients undergoing VIP, and 80% for the two patients undergoing TIP. The planned number of induction chemotherapy was achieved in 37 patients, with progression or death being the main reason for 18 patients who were not able to complete the planned number of first-line chemotherapy sessions. Furthermore, of the 55 patients, eight patients received chemotherapy dose reductions, and 13 patients experienced delayed administrations in at least one cycle of first-line chemotherapy.

Among the 45 patients with mediastinal NSGCT, no patient was classified into complete responses after first-line chemotherapy. A partial response with negative markers was seen in 24.4%, a partial response with positive markers was observed in 20%, 37.8% had stable disease, and the disease progressed for 17.8%.

Only six patients did not receive chemotherapy. These included three patients with NSGCT who received palliative and support care as they were admitted with an advanced oncologic disease as well as three patients with pure mature teratoma treated with surgical resection. Twenty-four patients underwent tumor resection surgery, of which 21 had surgery after completing the chemotherapy. Five patients with SGCT and 19 patients with NSGCT underwent post-chemotherapy surgery (**Table 2**). All patients with SGCT who underwent surgery had a 4-cm or greater residual mediastinal mass after first-line chemotherapy. However, no viable tumor was observed in the histopathologic report in any of these patients. The median diameter of the residual mediastinal mass in SGCT patients who underwent surgery was 6 cm (range, 4–8 cm).

**Table 2:** Treatment characteristics.

Characteristic	*n* (%)
Surgery	24 (39.3)
Complete resection	19 (79.1)
Incomplete resection	5 (20.8)
Surgical approach	
Clamshell	10 (41.6)
Median sternotomy	9 (37.5)
Other^a^	5 (20.8)
Histology according to surgery	24 (39.3)
NSGCT	19 (79.1)
SGCT	5 (20.8)
Tumor viability in surgical specimen	
NSGCT	16 (84)
SGCT	0 (0)
Postoperative complications	8 (33.3)
Hemorrhagic shock	3 (37.5)
Pneumonia and septic shock	2 (25)
Hemothorax	1 (12.5)
Surgical site infection	1 (12.5)
Myopericarditis	1 (12.5)
First-line chemotherapy	55 (90.2)
BEP (bleomycin, etoposide, cisplatin)	45 (81.8)
VIP (etoposide, ifosfamide, cisplatin)	6 (10.9)
TIP (paclitaxel, ifosfamide, cisplatin)	2 (3.63)
Carboplatin, etoposide, bleomycin	1 (1.81)
Monotherapy with etoposide	1 (1.81)
Second-line chemotherapy	16 (24.5)
VeIP (vinblastine, ifosfamide, etoposide)	6
VAC (vincristine, doxorubicin,	2
cyclophosphamide)	
Other^b^	8
Third-line chemotherapy	3 (4.9)
TIP (paclitaxel, ifosfamide, cisplatin)	2
GOP (gemcitabine, oxaliplatin, paclitaxel)	1

a Thoracotomy in 2 patients, thoracoscopy in 1 patient, and robotic surgery in 1 patient.

b Other chemotherapy schemes included GEMOX (gemcitabine and oxaliplatin)—1 patient, BEP (bleomycin, etoposide, cisplatin)—1 patient, ICE (ifosfamide, carboplatin, etoposide)—1 patient, TIP (paclitaxel, ifosfamide, cisplatin)—1 patient, ifosfamide and cisplatin—1 patient, ifosfamide, vinblastine, and paclitaxel—1 patient, doxorubicin—1 patient, and high-dose chemotherapy and autologous stem cell transplant—1 patient.

Three patients had intraoperative complications: two cardiac arrhythmias that required medical management and one superior vena cava lesion that required suture. No intraoperative mortality was observed. Two patients presented with postoperative pulmonary complications. Both had pneumonia and required prolonged endotracheal intubation; one of them died within the first month. Both patients were exposed to bleomycin before surgery. Four patients received adjuvant chemotherapy where etoposide and cisplatin regimen was administered in three patients and VIP in the other patient.

In our study, 19 patients with NSGCT presented relapse after first-line management, and 15 patients received conventional second-line chemotherapy. Vinblastine, ifosfamide, and cisplatin regimen was prescribed in six (9.8%) patients; two patients received vincristine, doxorubicin, and cyclophosphamide therapy due to findings of teratoma with malignant differentiation to rhabdomyosarcoma (**Table 2**). Only one patient from our cohort underwent high-dose chemotherapy and autologous stem cell transplant. This patient received this treatment as a second-line therapy and died due to infectious complications during the transplant.

### Survival Outcomes

The median time of follow-up was 11.7 months (IQR: 4.2–43.2). The median survival for the whole group was of 14.6 months (95% CI: 4.9–24.3 months), and the three-year cumulative survival rate was 38%. All the patients with primary mediastinal NSGCT were considered poor risk based on the International Germ Cell Cancer Collaborative Group (IGCCCG) classification, while six of 10 patients with mediastinal SGCT were classified as good risk, and four had intermediate risk.

**Figure 3:**
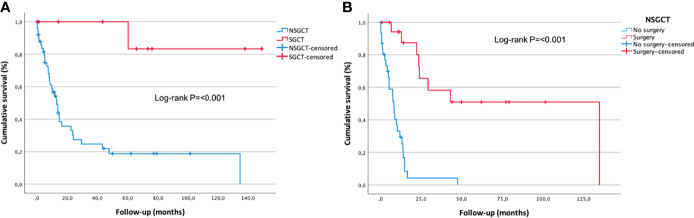
**(A)** Pure seminomatous germ cell tumor and non-seminomatous germ cell tumor overall survival. **(B)** Surgical and non-surgical management overall survival. Kaplan–Meier curve with log-rank test.

Patients with NSGCT had a median survival of 13 months (95% CI: 8.9–17.1 months), and the 3-year cumulative survival rate was 26%. In contrast, the median survival was not reached in patients with mediastinal SGCT (95% CI could not be calculated), and the three-year cumulative survival rate was 100% (**Figure 3A**). The median survival of NSGCT patients who underwent surgical resection of the mediastinal mass after chemotherapy (*n* = 19) was also not reached, and the 3-year cumulative survival rate was 59%. The subjects with NSGCT who did not undergo surgical management had a median survival of 8 months (95% CI: 6.5–9.5 months), while the 1- and 3-year cumulative survival rates were 33 and 7%, respectively (**Figure 3B**).

The chi-square test results revealed an association between death in patients with NSGCT and abnormal post-chemotherapy AFP (*p* = 0.05), non-surgical management (*p* = 0.002), and progression after first-line chemotherapy (*p* = 0.003). An association between progression in patients with NSGCT and abnormal initial AFP was also found (*p* = 0.013) (**Table 3**). The Cox proportional hazard model showed that patients with NSGCT who underwent surgical management of residual mediastinal mass after chemotherapy are less likely to die (HR: 0.029; 95% CI: 0.003 – 0.299, *p* = 0.003) and are less likely to have a progression (HR: 0.036; 95% CI: 0.003 – 0.375, *p* = 0.005).

**Table 3:** Chi-square test analysis for non-seminomatous germ cell tumors overall survival and progression-free survival.

Variable	No death	Death	No progression *n* = 29 (56.9%)	Progression	Chi-square *p*-value
	*n* (%) 16 (31.4)	*n* (%) 35 (68.6)		*n* = 22 (43.1%)	
Post-chemotherapy alpha-fetoprotein (abnormal)	3 (18.7)	16 (45.7)			0.050
Progression	2 (12.5)	20 (57.1)			0.003
Surgical management after chemotherapy	11 (68.7)	8 (22.8)			0.002
R0 surgery	10 (62.5)	4 (11.4)			0.111^a^
Initial alpha-fetoprotein (abnormal)			16 (55.2)	19 (86.4)	0.013
Post-chemotherapy lactate dehydrogenase (abnormal)			3 (10.3)	13 (59.1)	0.053
R0 surgery			11 (37.9)	8 (36.4)	0.111^a^

a Fisher’s exact test P-value.

## Discussion

In the most recent actualization from the IGCCCG, survival and prognostic factors were studied in 4,955 patients with NSGCT, of which only 182 patients (3.7%) had a mediastinal primary tumor (10). Even though a comparison with previous estimations in a 5-year follow-up shows an improvement in OS of up to 67% (95% CI: 65–69%) for poor-risk patients, primary mediastinal tumor still represents one of the most potent adverse prognostic factors for tumor progression (HR: 2.68, 95% CI: 2.04–3.53).

The experience from large reference cancer centers around the world is consistent with the expected poor survival of patients with mediastinal NSGCT. Bokemeyer et al. described an international analysis of 287 patients with mediastinal NSGCT, showing a 5-year survival of 45%, lower than retroperitoneal or testicular primaries (4). Rodney et al. published a 41% survival in a 51.3-month follow-up in 27 patients with mediastinal NSGCT (11). The median survival for patients with mediastinal NSGCT in the present study was 13 months, with a 3-year cumulative survival rate of 26%. These findings are inferior to the estimates mentioned above. We believe that the reason for this is a high incidence of SVCS and extrapulmonary metastatic involvement in the study participants, which is higher when compared to the other series reported in the literature (12, 13). Many of our patients had advanced disease at the time of diagnosis, with some not being able to receive appropriate multimodal management. A separate analysis of our patients with NSGCT who received multimodal treatment with perioperative chemotherapy and mediastinal resection surgery revealed outcomes similar to those reported from reference cancer centers worldwide for a 3-year cumulative survival rate (59%).

Our mediastinal GCT cohort is one of the largest reported in Latin America to date. However, other groups from the region have reported similar experiences. Mainieri-Hidalgo et al. described a 5-year survival of 58% in 19 patients from Costa Rica after initial therapy (14). In Argentina, Belen-Basile et al. reported the experience from 16 patients treated surgically, with a survival of 75% after a median follow-up of 36 months. Interestingly, there were seven female patients in this cohort, contrary to the male predominance reported for this disease (15).

The biologically aggressive behavior, the presence of histologic variants resistant to chemotherapy, and even the concomitant presence of hematologic neoplasms constitute additional reasons that may explain the poor outcomes for mediastinal NSGCT (6, 16). In our cohort, we observed five patients with postoperative histology compatible with teratoma with malignant differentiation. This histology is considered chemo-resistant and associated with adverse outcomes. Regarding concomitant hematologic malignancies, even though an incidence of up to 2% has been reported in the literature in patients with mediastinal NSGCT (16), we did not observe any in our cohort.

The operative outcomes from our study, one death (4.1%) and eight complications (33%), were consistent with previous reports. Kesler et al. (6, 17, 18) have described the biggest series of mediastinal NSGCT patients who underwent surgical resection after chemotherapy. In an initial report with 158 patients who underwent surgery (6), 10 postoperative deaths were observed (6%), and 26 patients (18%) had postoperative complications. In a posterior actualization with 255 patients who underwent surgery (18), 27 patients (10.9%) presented with respiratory failure, which was responsible for 11 (4.3%) deaths. They also found that 25/27 patients with respiratory failure were exposed to bleomycin prior to surgery (*p* = 0.004). In our experience, two patients had prolonged endotracheal intubation in the postoperative period, and both were exposed to bleomycin. Even though the sample size in our study and the number of outcomes did not allow us to show an association between postoperative respiratory failure and previous bleomycin exposure, a biological plausibility exists, and our findings were consistent with the data reported by Kesler et al. (6, 17, 18). Although other groups could not demonstrate an increased respiratory failure risk with perioperative bleomycin (19), it is reasonable to choose chemotherapy schemes without this agent to treat mediastinal NSGCT patients. In 2018, we changed the initial chemotherapy regimen in our treatment protocol for mediastinal NSGCT. As can be seen in **Figure 2**, we offer VIP as first-line chemotherapy given the evidence of similar outcomes when compared with BEP (20).

In our cohort, we observed an association between some variables (abnormal post-chemotherapy AFP, tumor relapse after primary therapy, non-surgical management, and non-seminomatous histology) and an adverse survival prognosis. All these variables have been consistently linked in the literature as prognostic factors in patients with mediastinal GCT (6, 12, 13, 21–24).

The prognosis of relapsing patients after first-line treatment is ominous. High-dose chemotherapy with autologous stem cell transplant is an attractive alternative for management (25–27). However, the results from a phase III clinical trial are expected to define whether this is standard of care (28). In our cohort, only one relapsing patient underwent this therapy. More research efforts must be channeled in the pursuit of new treatment strategies to improve the survival of these patients.

SGCT represents a biologically different disease compared to NSGCT. These neoplasms usually have a higher response probability to first-line chemotherapy and a better survival prognosis compared to NSGCT. Multiple publications show how patients with pure seminomatous tumors achieve a 5-year survival of over 90% when adequate cisplatin-based chemotherapy is used (5, 29). In our study, we observed a 3-year survival of 100% in the patients with mediastinal SGCT.

The limitations of this study include its retrospective nature and the single-center analysis. Given the small sample size, we could not demonstrate a statistical association between OS and important variables such as tumor viability in the surgical specimen or incomplete tumor resection, variables linked in the literature to worse survival prognosis in NSGCT patients. The high percentage of patients with lost serum tumor marker data at the beginning of the treatment also limited the multivariate survival analysis. Second-line chemotherapy was inconsistent due to the use of multiple regimens and poor access to high-dose chemotherapy with stem cell rescue.

## Conclusions

We present one of the largest mediastinal GCT series in South America, with survival outcomes similar to other lower- and median-income countries. Mediastinal NSGCT are rare neoplasms with poor oncological prognosis; multi-modality management with first-line chemotherapy and surgery of any residual mediastinal mass offers the best chances of remission and long-term survival to these patients. The variables associated with worse survival prognosis in mediastinal NSGCT in our study include abnormal post-chemotherapy AFP, tumor relapse after primary therapy, and non-surgical management. Mediastinal SGCT are neoplasms with a less aggressive biological behavior and with higher survival chances compared to NSGCT.

## Data Availability Statement

The original contributions presented in the study are included in the article/supplementary material. Further inquiries can be directed to the corresponding author.

## Ethics Statement

The studies involving human participants were reviewed and approved by the Institutional Ethics and Research Committee of the National Cancer Institute (no. CEI-00526-20). Written informed consent from the legal guardian/next of kin of the participants was not required in this study in accordance with national legislation and institutional requirements.

## Author Contributions

CV-Y, CC-F, RB-M, JB, RB, and RB-J contributed to study conception and design. CV-Y and CC-F contributed to the collection of clinical data. CC-F and JC-D contributed to statistical analysis. CV-Y, CC-F, and RB-M drafted the manuscript. All authors contributed to the article and approved the submitted version.

## Funding

This work was supported by the National Cancer Institute in Bogota, Colombia.

## Conflict of Interest

The authors declare that the research was conducted in the absence of any commercial or financial relationships that could be construed as a potential conflict of interest.

## Publisher’s Note

All claims expressed in this article are solely those of the authors and do not necessarily represent those of their affiliated organizations, or those of the publisher, the editors and the reviewers. Any product that may be evaluated in this article, or claim that may be made by its manufacturer, is not guaranteed or endorsed by the publisher.
